# Ocular Delivery of Polyphenols: Meeting the Unmet Needs

**DOI:** 10.3390/molecules26020370

**Published:** 2021-01-12

**Authors:** Luna Krstić, María J. González-García, Yolanda Diebold

**Affiliations:** 1 Insituto de Oftalmobiología Aplicada (IOBA), Universidad de Valladolid, 47011 Valladolid, Spain; lkrstic@ioba.med.uva.es (L.K.); mjgonzalez.ioba@gmail.com (M.J.G.-G.); 2 Centro de Investigación Biomédica en Red Bioingeniería, Biomateriales y Nanomedicina (CIBER-BBN), Instituto de Salud Carlos III, 28029 Madrid, Spain

**Keywords:** polyphenols, ocular drug delivery, oxidative stress, inflammation

## Abstract

Nature has become one of the main sources of exploration for researchers that search for new potential molecules to be used in therapy. Polyphenols are emerging as a class of compounds that have attracted the attention of pharmaceutical and biomedical scientists. Thanks to their structural peculiarities, polyphenolic compounds are characterized as good scavengers of free radical species. This, among other medicinal effects, permits them to interfere with different molecular pathways that are involved in the inflammatory process. Unfortunately, many compounds of this class possess low solubility in aqueous solvents and low stability. Ocular pathologies are spread worldwide. It is estimated that every individual at least once in their lifetime experiences some kind of eye disorder. Oxidative stress or inflammatory processes are the basic etiological mechanisms of many ocular pathologies. A variety of polyphenolic compounds have been proved to be efficient in suppressing some of the indicators of these pathologies in in vitro and in vivo models. Further application of polyphenolic compounds in ocular therapy lacks an adequate formulation approach. Therefore, more emphasis should be put in advanced delivery strategies that will overcome the limits of the delivery site as well as the ones related to the polyphenols in use. This review analyzes different drug delivery strategies that are employed for the formulation of polyphenolic compounds when used to treat ocular pathologies related to oxidative stress and inflammation.

## 1. Introduction: Polyphenols a Remedy from Nature

Polyphenols represent one of the most abundant groups of phytochemicals present in nature; indeed, more than 10,000 different compounds can be found throughout the plant kingdom [1]. As secondary metabolites, they have different roles, such as prevention of pathogen development and protection from UV radiation. Beside these, polyphenols offer protection from excessive development of reactive oxygen species (ROS), herbivores, and environmental stresses. Additionally, they are involved in physiological functions that are essential for the well-being of the plant. Higher resistance to predators, pigmentation, lignification, or increased astringency of fruit are some of these functions [2,3].

**Figure 1:**
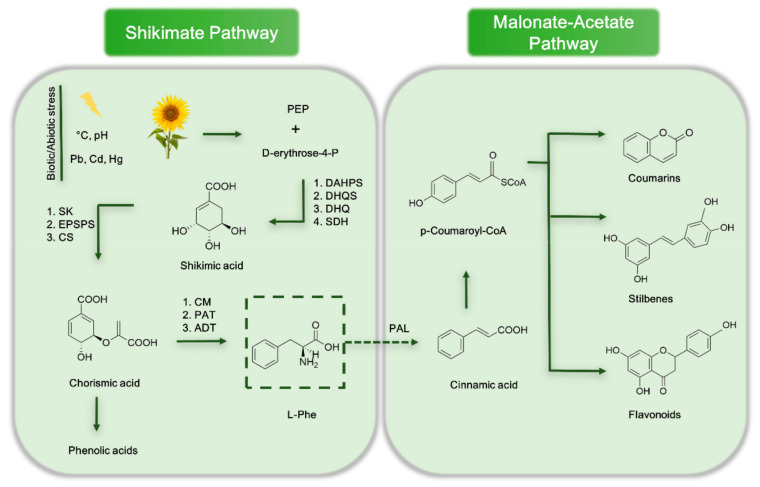
Schematic showing of the shikimate and malonate-acetate pathway in plants. Note: DAHPS—3-deoxy-d-arabino-heptulosonate-7-phosphate synthase; DHQS—3-dehydroquinate synthase; DHQ—3-dehydroquinate dehydratase; SDH—shikimate dehydrogenase; SK-shikimate kinase; EPSPS—5-enolpyruvylshikimate 3-phosphate synthase; CS—chorismate synthase; CM—chorismate mutase; PAT—prephenate aminotransferase; ADT—arogenate dehydratase; PAL—phenylalanine ammonia-lyase; PEP—phosphoenolpyruvic acid.

There are two main synthetic pathways through which polyphenols are built in plants, the shikimate and the malonate-acetate pathway [4]. The first one comprises seven consecutive enzymatic steps that are carried out in the chloroplast. Activation of this synthetic pathway is promoted by different biotic and abiotic factors; these include adverse temperature and pH, the presence of pathogens and herbivores, and stress produced by an excessive concentration of heavy metals or salts [5]. In the second pathway, the phenolic ring structure is formed under the successive addition of malonyl coenzyme A moieties to the polyketide chain; these sequential reactions characterize the malonate-acetate pathway that is mostly exploited by the plants for the synthesis of different sub-classes of flavonoids, such as flavones, isoflavones, or flavanones [6]. Figure 1 illustrates the two synthetic pathways of polyphenols in plants.

Due to their involvement in different molecular pathways and the possession of different healing properties towards a vast range of diseases, polyphenolic compounds have acquired the interest of researchers from different fields. These abilities are supported by various in vitro and in vivo studies, as well as the clinical ones [7].

### 1.1. Classification of Polyphenols

**Figure 2:**
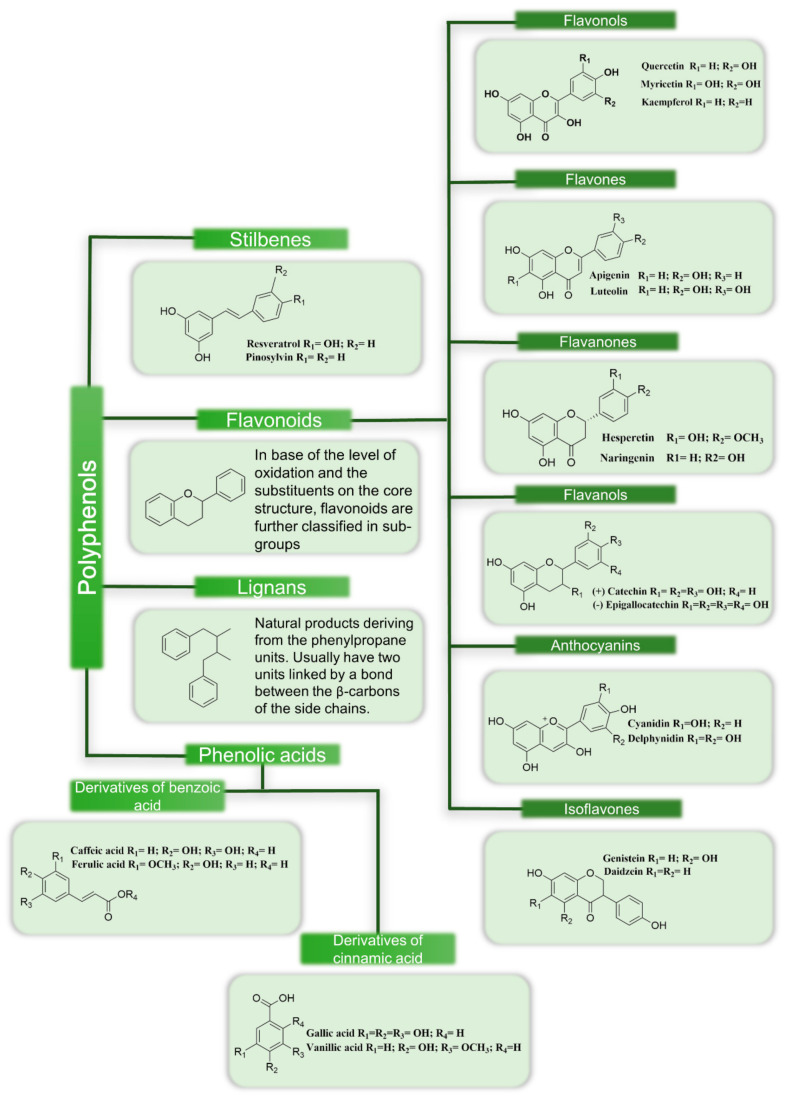
Schematic showing the classification of polyphenols.

Based on their structural complexity polyphenols can be classified in different groups and subgroups. For example, certain structural patterns are typical for specific groups of polyphenols, such as the C_6_-C_3_-C_6_ pattern (two aromatic rings interconnected with an oxygen bearing heterocycle) representative of flavonoids [8]. According to the level of oxidation of the central ring, flavonoids are further classified in six subgroups: flavonols, flavones, flavanones, flavanols, anthocyanins and isoflavones. Non-flavonoid polyphenols include the classes of stilbenes, phenolic acids, and lignans. Among these latter classes typical chemical “fingerprints” are found; two benzene rings joined by a methylene bridge (C_6_-C_2_-C_6_) are characteristic of stilbenes, while for lignans, the union of two phenylpropanoid moieties (C_6_-C_3_) is typical, and at β and β’ carbon atoms, these moieties can be joined to an ether, lactone, etc. [9]. Figure 2 schematically illustrates the classification of polyphenols.

### 1.2. Natural Sources of Polyphenols

The ubiquity of polyphenolic compounds in nature is confirmed by their presence not only in fruits and vegetables, but also in whole grain cereals, coffee, tea, and red wine [10]. Plant sources of polyphenols differ by the quantity and the variety of the polyphenolic compounds that they possess due to different factors to which these sources can be exposed. For example, environmental factors, such as soil content, sun exposure, or even the ripeness of the fruit, influence the final concentration of the polyphenols. Various studies have reported that even the storage conditions or the method of culinary preparation have a significant effect on the polyphenolic content [11].

Flavonols, the most predominant sub-class of flavonoids, are mainly found in leafy vegetables such as lettuce and cabbage, onions, broccoli, and blueberries [12]. These compounds usually accumulate in the part of the plants that are more exposed to sunlight, since their biosynthesis is activated by light. The most abundant flavonols are quercetin and kaempferol. Celery and parsley represent a source of luteolin and apigenin, which are flavones, a less common sub-class of flavonoids. Daidzein and genistein, mostly found in leguminous plants, such as soya, are isoflavones [13], are usually considered as phytoestrogens due to the high structural similarity with human estrogens. Flavanones such as hesperetin or naringenin are detected in citrus fruit, such as oranges or lemons, while chocolate and green tea are the richest sources of flavanols [12,14]. The latter ones can exist in both monomeric and polymeric form. Pigments, such as anthocyanins, are mostly found in fruits, usually complexed with other flavonoids, and this complexation exerts a stabilization effect on them.

The derivatives of benzoic acid and those of cinnamic acid are distinguished among other phenolic acids. The derivatives of benzoic acid, in their free form or esterified, are present in low quantities in only few edible plants and are, therefore, not considered of major nutritional and health interest. On the contrary, the derivatives of cinnamic acid, which are usually found in glycosylated or esterified forms, are more common, and fruits such as plums, kiwis, and blueberries have the highest content [11].

Resveratrol, a stilbene, and the compound that is responsible for the well-known French paradox (a lower incidence of cardiovascular diseases despite a high fat diet related to the supposed high consumption of wine by French people), is found mostly in red wine [15]. Oleaginous seeds have been identified as the primary source of lignans, while onions, asparagus, wheat, and pears have been identified as secondary sources [11,16].

It is interesting to mention that in addition to terrestrial plants, polyphenols are found in marine microalgae. Compounds such as benzoic acid and vanillic acid are extracted from the genus *Gracilaria*, while isoflavones such as daidzein or genistein are extracted from red and brown seaweeds [17,18].

## 2. The Eye: A Sensory Organ Susceptible to External Stress

The eye is a unique sensory organ, with complex anatomical and physiological features. As a unit, it can be divided into two main segments: the anterior segment, which comprises the cornea, conjunctiva, iris, ciliary body, and the lens, and the posterior segment, which includes the choroid, retina, and sclera. The light that enters the eye is refracted by the cornea, then proceeds through the lens, and finally reaches the retina, which, once the photoreceptor cells are activated, enables a signal transduction to the brain and the generation of an image [19,20,21]. The direct contact of the eye with the external environment makes it persistently exposed to sunlight, but also to other sources of ROS, such as environmental toxins, ionizing radiation, smoke, and different atmospheric pollutants. It has to be added that different ocular cell types possess a high content of mitochondria, where oxidative phosphorylation takes place. For instance, the photoreceptors present in the retina have a high rate of aerobic ATP production, which induces a high rate of ROS production as a direct consequence. All this make the eye particularly vulnerable to oxidative stress [22,23].

### 2.1. Oxidative Stress

**Figure 3:**
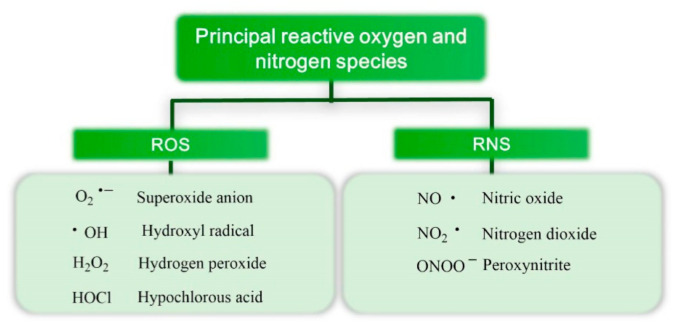
Principal reactive oxygen species (ROS) and reactive nitrogen species (RNS) present in the human body.

Free radicals are a physiological product of our metabolic reactions and are required for the correct preservation of such functions as signal transduction, gene expression, and cell proliferation. This explains why some of the main sources of ROS and reactive nitrogen species (RNS) are vital cellular mechanisms. The main ROS and RNS present in the human body are illustrated in Figure 3.

There are different examples of mechanisms that include the generation of free radicals. These comprise the respiratory chain that occurs in the mitochondria or the catalytic activity of some enzymes. Such good examples are represented by the activities of xanthine oxidase or nitric oxide synthases (NOS), which catalyzes the conversion of L-arginine into nitric oxide (NO) [24,25]. For example, in the cornea, ROS generation is a direct consequence of its permanent exposure to UV radiation [26]. 

In some cases, the fine enzymatic machinery that regulates the equilibrium between the production and elimination of ROS and RNS is compromised, which results in an uncontrolled production of radical species that can damage vital macromolecules. This damage leads to the onset of different pathological conditions, such as cardiovascular diseases, tumorigenesis, neurodegenerative diseases, and diabetes [26,27,28,29,30]. 

The continuous exposure of human tissues and organs to distinct sources of oxidative stress has led to the development of several protection “mechanisms” that act as a counterbalance to the extensive presence of this type of stress. These mechanisms are located in both intra- and extracellular spaces and can be part of enzymatic systems, chelating agents, or free radical scavenger molecules. Examples of antioxidant enzymes in the eye are superoxide dismutase (SOD), catalase and glutathione peroxidases, and reductases. These enzymes work in association. SOD, which is present in different isoforms, catalyzes the conversion of the superoxide radical into hydrogen peroxide. On the other hand, catalase has a double function since it protects the eye from hydrogen peroxide, but it also prevents the inactivation of SOD. Glutathione peroxidases and reductases regulate the transformation of organic peroxides into water or alcohols. As regards nonenzymatic antioxidants, the two most important are vitamin C and glutathione. The highest levels of these substances are found in the corneal epithelium and in the lens, respectively. This demonstrates that vitamin C and glutathione have an important role in preserving the structures of the anterior part of the eye from oxidative stress.

The fine controlled antioxidant activity that limits further propagation of the oxidative stress decreases with aging, so the emergence of pathologies is more likely to occur at advanced ages. Therefore, increased attention is being paid to supplementation with exogenous antioxidant molecules of plant origin and their use in the treatment/prevention of diseases related to oxidative stress [31,32,33,34,35]. 

### 2.2. Inflammation

Inflammation represents one of the most important biological mechanisms of tissue defense against microorganism invasion and mechanical or chemical tissue injuries [36]. If these injuries are of a mild form, the inflammation response is acute. Persistence of the injuries may lead to a chronic inflammatory process that eventually becomes pathological. 

The inflammatory process appears to be complex because it is regulated by a diversity of molecular pathways. The activation of mediators such as tumor necrosis factor alpha (TNF-α), cyclooxygenase-2 (COX-2), or inducible nitric oxide synthase (iNOS) is modulated by the nuclear factor kappa-light-chain-enhancer of activated B cells (NF-kB) signaling pathway. Signal transducer and activator of transcription 3 (STAT3) is a transcription factor that participates in the inflammatory cascade by regulating the expression of different pro-inflammatory cytokines. The nuclear translocation of STAT3 is induced by phosphorylation by a Janus-activated kinase (JAK). Protein kinases p38, c-Jun N-terminal kinase (JNK), and extracellular signal-regulated kinases (ERK), from the family of mitogen-activated kinases (MAPK), have also been found to control the levels of different cytokines, such as interleukin-5 (IL-5). Besides these other important mediators of the inflammatory process, there are transcription factors such as nuclear factor of activated T cells (NFAT), nuclear factor erythroid-2 related factor 2 (Nrf2), and activator protein-1 (AP-1) [37,38].

The eye is an important sensory organ that can be particularly endangered by the inflammatory process. Therefore, it is not unusual that the origin of many ocular disorders lies in this process. An adequate therapeutic approach can result in successful management of the inflammation, and thus, limit the possibility of the development of side effects that can lead to vision impairment.

The eye functions as a separate unit since it is protected from molecules circulating in the peripheral blood system through the blood-aqueous and blood-retinal barriers. Under ocular inflammatory conditions, the structural integrity of these barriers can be compromised, resulting in the further tissue infiltration of immune cells and the production of high levels of inflammatory mediators [39,40,41].

## 3. Ocular Pathologies Related to Oxidative Stress and Inflammation

Oxidative stress and inflammation have been reported to be implied in the pathophysiological mechanism of many diseases of the eye. Dry eye disease, cataracts, glaucoma, age-related macular degeneration, and diabetic retinopathy are examples of such diseases. These diseases are discussed in more detail in the following subsections.

### 3.1. Dry Eye Disease

Dry Eye Disease (DED) is a multifactorial disease of the ocular surface that affects around 30% of the population worldwide over the age of 50 [42]. It is distinguished by the loss of the homeostasis in the tear film as well as by tear film instability and hyperosmolarity, inflammation and damage of the ocular surface, and neurosensory abnormalities [43]. ROS plays a key role in the diffusion of the dry eye cycle: stress signaling pathways that are induced upon excessive ROS content in tears and ocular surface lead to inflammation and succeeding infiltration of the immune cells, which directly leads to an abnormal tear film [44]. The important role of inflammation in the evolution of DED was confirmed by the analysis of tears of DED patients, where an overexpression of various pro-inflammatory cytokines and chemokines, such as TNF-α, interleukin 1 receptor antagonist (IL-1RA), interleukin 1 (IL-1), interleukin 6 (IL-6), and interferon gamma-induced protein 10 (IP-10), was observed [45].

The current therapeutic approach for the treatment of DED consists of the use of artificial tears and/or eyelid hygiene. Management of the inflammation of the ocular surface is done by the administration of topical corticosteroids. Their prolonged use can lead to possible serious side effects, such as the development of glaucoma or cataracts. An alternative to corticosteroids could be the use of Cyclosporine A, a cyclic undecapeptide. Despite being a very popular line of treatment for patients with DED, optimal doses and the optimal duration of treatment are still debatable. Furthermore, adverse effects such as foreign body sensation, conjunctival hyperemia, and epiphora can accompany treatment with this medicine [46,47,48]. Another alternative could be lifitegrast. Lifitegrast is a small molecule whose antagonistic activity inhibits the propagation of inflammation by preventing interactions between lymphocyte function-associated antigen (LFA-1) and intercellular adhesion molecule 1 (ICAM-1) [49]. Treatment with this drug is accompanied by side effects that include irritation of the site of instillation, blurred vision, and discomfort [50].

There is a need for novel therapeutic approaches for DED. One of these surely could be in the application of compounds of a natural origin. Compounds such as curcumin or quercetin have already exhibited encouraging results in both in vitro and in vivo models [51]. Improvement of marketed formulations with the addition of polyphenols, like in the case of Hypromelóza-P^®^ and fisetin, could also be a promising strategy [52].

### 3.2. Cataracts

A cataract, a condition of the opacification of the eye lens, is a pathology that affects more than 68% of people over the age of 79 worldwide [53]. The light that enters the eye is focused by the lens on the retina and, therefore, must remain transparent [54]. Changes in the conformation of crystallins, the main proteins in the lens, lead to their aggregation, causing an alteration in the lens refractive index. The conformational changes are a consequence of high levels of ROS accumulated by years of metabolic reactions. At present, cataracts are cured by surgical interventions to replace the opaque lens with an intraocular lens.

Various molecules of plant origin have been suggested as possible methods of cataract treatment [55,56]. For instance, the extract from the leaves of *Ginkgo biloba* have shown to be efficient in preventing the inducement of cataracts via its antioxidant property. The same property has been ascribed to lutein and zeaxanthin. Luteolin, a flavonoid compound, can also inhibit ROS formation and prevent lipid peroxidation in the lens. Additionally, attenuation in cataract formation has been ascribed to curcumin [55].

### 3.3. Glaucoma

Glaucoma is identified as one of the major causes of blindness worldwide and it is estimated to affect more than 111 million people in 2040 [57]. Glaucoma groups several progressive neuropathies characterized by the degeneration of retinal ganglion cells and changes in the optic nerve head. It has been proved that aging and increased intraocular pressure (IOP) play an important role in the evolution of the disease. In addition to these, oxidative stress, inflammation, hypoxia, and vascular impairment are recognized as part of the pathogenic mechanism of glaucoma [58,59].

Current therapies include the chronic administration of drugs that are specific for the lowering of intraocular pressure. These include different classes of molecules, such as carbonic anhydrase inhibitors, prostaglandin analogues, and β-adrenergic blockers. Conjunctival hyperemia, uveitis, burning, and irritated eyes are only a few of the undesired effects that follow the administration of this kind of therapy. To date, this is the only effective method for treating glaucoma, although, in more advanced stages of the diseases, surgical methods are necessary [57].

There is an urgent need for new therapeutic approaches in the treatment of glaucoma, especially focused on the protection of ganglion cells from apoptosis. Application of antioxidant molecules could represent a future line of medications. Some natural antioxidant molecules have been proven to be effective in in vitro studies. For example, resveratrol was able to protect primary porcine trabecular meshwork cells subjected to oxidative stress from apoptosis [60]. Another study has highlighted the beneficial effects of resveratrol in protecting retinal ganglion cells in rats from retinal ischemia induced by high intraocular pressure [61]. *Ginkgo biloba* has been found to be effective in increase the ocular blood flow in normal tension glaucoma patients [60]. iTRAB^®^, a commercially available formulation containing highly concentrated polyphenols and fatty acids, was able to reduce oxidative stress and the inflammatory response in a 3D model of Human Trabecular Meshwork Cells [62].

### 3.4. Age-Related Macular Degeneration

Age-Related Macular Degeneration (AMD) is characterized by the destruction of retinal layers and the accumulation of lipids and proteins in the macula, the central region of the retina. As previously mentioned, different factors make the retina particularly susceptible to oxidative stress; these include not just the direct exposure to light and a high rate of oxygen metabolism, but also the presence of highly photosensitive molecules, such as lipofuscin, which can act as a generator of free radical species [63,64]. Vascular factors, whose dysregulation leads to AMD, can be compromised not only by ROS but also by hypoxic conditions. The latter promote the expression of hypoxia inducible factor-1 (HIF-1) and vascular endothelial growth factor (VEGF), both of which promote the development of new blood vessels [65].

Current treatments of AMD involve the use of monoclonal antibodies, whose administration is performed through intravitreal injections, an administration method that increases the risk of development of ocular infections and has a low patient compliance. Additionally, this kind of therapy is reported to be expensive and not accessible to everyone [66,67].

Therefore, there is a need for the development of alternative therapeutic approaches with reduced side effects. Several reports have shown that the employment of naturally occurring compounds, such as curcumin, resveratrol, quercetin, and epigallocatechin, can reduce the development AMD [68,69]. Since these polyphenols offer efficacious protection against oxidative stress, which is highly implied in the development of this ocular disease, greater efforts should be made in the potential therapeutic use of some of these compounds.

### 3.5. Diabetic Retinopathy

Diabetic retinopathy, one of the major consequences of diabetes, is a low advancing chronic disease that, at its early stages, causes vision impairment and in advanced stages, if not treated properly, can lead to vision loss [70]. It is estimated that, by 2030, the number of people affected worldwide by diabetic retinopathy (DR) will be around 191 million [71]. At its early phase, the disease is characterized by microaneurysms and small dot-like hemorrhages, which increase in size and number as the disease progresses. At a developed stage, the formation of a novel vasculature departing from retinal blood vessels occurs. Visual loss is a result of the hemorrhages caused by these newly formed vessels [72]. 

Diabetic hyperglycemia leads to alteration in different metabolic pathways, including the activation of the polyol and hexosamine pathways, an increment in the production of advanced glycation end products (AGEs), a higher expression of the related receptors, and the setting off of the pathway related to protein kinase C (PKC). The activation of these biochemical pathways leads to an excessive production of ROS and subsequently to oxidative stress. Another enzymatic system that contributes to the generation of oxidative stress is the one related to the family of NADPH oxidases (Nox). This system incorporates different isoforms, of which Nox 1, Nox 2, and Nox 4 are highly expressed inside the vascular system. This results in promoting retinal neovascularization through the expression of vascular endothelial growth factor (VEGF), whose activation is ROS-dependent [73,74,75].

There are now different therapeutic approaches to managing DR. These approaches include laser photocoagulation, the administration of corticosteroids, vitreoretinal surgery, and the intravitreal injection of anti-VEGF. Even though these therapies have proven to be helpful in treating DR, they are not easily accessible because of their high cost. This is not the only limitation of traditional therapeutic approaches in treating DR; there are also side effects, such as retinal detachment and laser-induced burns in retinal tissue that may lead to blindness. Thus, besides these traditional approaches, novel therapeutic approaches with the use of naturally occurring compounds are a promising alternative [76,77,78,79]. Compounds such as quercetin, epigallocatechin gallate, and hesperetin have been shown to improve retinal oxidation levels in rats with diabetes induced by streptozotocin [80].

## 4. An Overview of Ocular Delivery Strategies for Polyphenols

It is estimated that around 258 million of people worldwide suffers from visual impairment. This makes the field of ophthalmic therapeutics particularly interesting for researchers [81]. Unfortunately, delivering therapeutics to the eye is exceedingly demanding. Because of the peculiar anatomical and physiological characteristics that the eye possesses. Anatomical barriers include such structures as the corneal epithelium and the blood ocular barriers, and physiological barriers comprise processes such as the tear fluid turnover, nasolacrimal drainage, and blinking [82,83]. For delivering therapeutic agents to the anterior part of the eye, a topical administration route is preferred. It is a non-invasive method of local application of the drug. However, the bioavailability of the required therapeutic agents is often <5%, which is an important limitation. Common topical formulations include eye drops, ointments, and emulsions [84]. Pathologies affecting the posterior part of the eye are even more difficult to treat. Treatment of these pathologies often involve invasive procedures, such as the implantation of drug-containing depots, surgeries, and intraocular injections [85,86]. Thus, there is a need to develop advanced delivery formulations that can overcome the hurdles that we face when treating ocular pathologies.

Polyphenols are interesting as potential therapeutics for the eye, as they are potent antioxidants, are abundant, are specific in interacting with distinct biological pathways, and possess low toxicity. However, the main obstacle for their broader use in therapeutics is related to their low bioavailability, which forces the use of higher concentrations [87]. A good example of this is represented by resveratrol, which has low solubility in water and is unstable, which negatively influences its bioavailability [88]. Therefore, they are suitable candidates for the employment of advanced methods of nanosized formulation approaches [89].

In the following subsections the characteristics of different phenolic compounds that are employed in the treatment of ophthalmic diseases as well as different formulation approaches are discussed. Table 1 summarizes the drug delivery systems used for the ophthalmic delivery of polyphenols covered in this review.

### 4.1. Resveratrol

Resveratrol was mentioned for the first time in 1939, when Takaoka published his article: “Resveratrol, a new phenolic compound, from Veratrum grandiflorum” [113]. Since then, the interest for this phenolic compound has grown exponentially: numerous scientific papers, book chapters, patents, symposiums, and conferences have been dedicated to it [114]. Resveratrol (3,5,4′-trihydroxy-trans-stilbene) is a phenolic compound belonging to the non-flavonoid group of stilbenes. The chemical structure of resveratrol comprises two aromatic rings that are joined by a methylene bridge, and this allows the existence of two isomeric forms of resveratrol the cis- and the trans-form. The trans-form is the predominant one, to which biological effects are attributed [115]. When trans-resveratrol is exposed to sunlight or artificial UV light at wavelengths of 254 nm or 366 nm, the interconversion to cis-resveratrol occurs [116]. External stimuli, such as UV light or pathogen invasion, stimulate the biosynthesis of resveratrol in plants. Resveratrol and other stilbene derivates are mostly found in those parts of the plants that are not photosynthetically active. Indeed, it has been shown that, in active ones, they restrict ion transport and redox reactions [117]. 

Resveratrol has gained attention over the years thanks to the anti-oxidative, anti-inflammatory, anti-apoptotic, and anti-angiogenic effects attributed, among others. It has been used in the treatments of cancer, cardiovascular diseases, neurological disorders, and diabetes, among others [118]. As regards ocular pathologies, resveratrol has shown its potential in the treatment of cataracts, glaucoma, and diabetic retinopathy [119]. 

Although resveratrol possesses beneficial health effects, its further use in therapeutics is limited by its pharmacokinetics: it is rapidly metabolized and expeditiously eliminated from the organism. In addition, its bioavailability is further limited by its low solubility in water. These problems have been addressed by the employment of advanced delivery systems, such as nanoparticles (NPs), micelles, liposomes, and inserts [120].

A study by Dong et al. evinced the efficacy of gold NPs coated with resveratrol against streptozotocin induced diabetic retinopathy in Wistar male rats. The authors have evaluated the effect of the formulation on different parameters such as the retinal vascular permeability, morphological variations in the retina, and expression of inflammatory cytokines and chemokines. They reported that vascular permeability upon the treatment with the formulation (300 mg/kg/day of Au-NPs) suffered a noticeable decrement (6.2 ± 1.2 ng/mg) when compared to that of the diabetic rats without treatment (13.2 ± 1.6 ng/mg). The levels of cytokines (IL-6 and IL-1β) and the retinal levels of Intercellular Adhesion Molecule-1 (ICAM-1) and Vascular Cell Adhesion Molecule (VCAM-1) were decreased by more than 30% upon treatment with the formulation [90]. 

Buosi et al. explored the encapsulation of resveratrol in a nanogel based on chitosan with a high molecular weight. To study the anti-inflammatory activity of the formulation ARPE-19 cells derived from human retinal pigment epithelium (RPE) were stimulated with lipopolysaccharide. No significant production of proinflammatory interleukins IL-6 and IL-8 was observed in cells that were treated with the formulation when compared to untreated control cells [91].

Bhatt et al. have proposed the use of poly(lactic-co-glycolic-acid) nanoparticles for the delivery of resveratrol. The NPs were efficient in reducing the expression of VEGF in ARPE-19 cells, and therefore, could be a useful strategy for the treatment of neovascular AMD [92].

### 4.2. Quercetin

Inside the class of flavonoids and polyphenols in general, quercetin (2-(3,4-dihydroxyphenyl)-3,5,7-trihydroxychromen-4-one) emerges as one of the most studied compounds, being a leading candidate for use in therapy. Its numerous favorable health effects are a direct consequence of its chemical structure. Two benzene rings, connected to an additional pyrone ring, represent the carbon skeletal with a typical C_6_-C_3_-C_6_ formation, characteristic of flavonoids. In addition, the carbon skeleton bears five hydroxyl groups; quercetin is also referred to as pentahydroxyflavonol [121,122]. Due to its high predisposition to degradation with the low solubility in water, quercetin has a low bioavailability. Therefore, there is a necessity of advanced formulation solutions for this molecule in order to be used in therapy [123,124].

Through the involvement in individual molecular pathways and mechanisms quercetin plays a promising role in the potential treatment of a multiplicity of diseases, such as: cardiovascular diseases, hypertension, Alzheimer’s disease, cancer, and allergies [125,126,127,128,129].

The pharmacological action of quercetin has been shown by the implication in different mechanisms that regulate cell homeostasis. For example, the anticancer effect of quercetin is provided by the inhibition of proteins that are involved in cell survival signaling such as protein kinase C (PKC-α), AKT, and ERK, and the setting off of cell death signals, such as PKC-δ or JNK. Quercetin is able to stimulate proapoptotic Bcl-2 proteins, such as Bax and Bad in multiple cell lines [130,131,132].

The anti-inflammatory effect provided by quercetin is a direct consequence of its capacity of scavenging free radical species, which promotes the NF-kB transcription factor. After activation under ROS, NF-kB translocates into the nucleus where it functions as a gene promoter that encodes for different pro-inflammatory cytokines and chemokines (IL-1, IL-6, TNF-α) [133]. There are several studies that have shown the anti-inflammatory effect of quercetin in vitro. Our group (Abengózar et al., 2015) showed that a concentration of quercetin of 20 μM or lower was able to reduce the levels of inflammatory cytokines IL-6, IL-8, and IP-10 when conjunctival epithelial cells were stimulated with TNF-α. Even lower levels of quercetin (≤5 μM) were enough to decrease the levels of the cytokines in corneal epithelial cells (HCE) [134]. Quercetin alone, and in combination with resveratrol, has been used to treat experimental DED induced in mice exposed to desiccating stress [93]. In this case, DED animals were treated with formulations of the two polyphenols with β-cyclodextrin. We confirmed that both topical quercetin or resveratrol or their combination reduces an inflammatory response in vivo by reducing the levels of IL-1α and by limiting the infiltration of CD4 + T cells into the conjunctival tissue of the murine DED model [93].

### 4.3. Epigallocatechin Gallate

The structure of epigallocatechin gallate (EGCG) comprises a gallate ester and a gallocatechol group bounded to a central flavanol unit [135]. It is thanks to these structural properties that EGCG is considered to be a powerful scavenger of free radical species, even more powerful than vitamin E [136]. EGCG has been reported to have beneficial effects in the treatment of cardiovascular pathologies, metabolic disorders, cancer, and neurodegenerative diseases, among others [137]. The mechanisms that are modulated by EGCG include the repression of MAPK, the inhibition of the expression of various transcription factors, such as AP-1 or NF-kB, modulations of proteins that regulate the cell cycle (cyclins), and reductions in the expression of HIF-1α [138,139]. 

The topical ocular delivery of EGCG was intended with the preparation of gelatin NPs that were decorated with hyaluronic acid. All of the components of the nanoparticles self-assembled thanks to the exploitation of electrostatic forces. This NP formulation was aimed to be used in the treatment of DED and showed promising results in both in vitro and in vivo experiments [94]. The expression of pro-inflammatory cytokines (IL-6, IL-8, and IL-1β) was significantly decreased in LPS-stimulated Human Corneal Epithelial Cells (HCEC) that were treated with EGCG-loaded gelatin NPs. Regarding in vivo experiments, improved tear secretion was observed in DED rabbits that were treated with formulation reaching levels close to that of normal control animals [94].

Endothelial cells express different proteins during ocular neovascularization, including α_v_β_3_ integrin. This protein can be targeted by a specific peptide sequence, arginine-glycine-aspartic acid (RGD peptide), which is used in drug delivery. This was the idea that was exploited by Chang et al. when designing a formulation aimed at treating corneal angiogenesis. The formulation consisted of gelatin NPs coated with the RGD sequence and HA and loaded with EGCG [95]. The formulation was able to recognize the integrin of interest, which was expressed by human umbilical vein endothelial cells (HUVEC). The inhibition of migration in formulation-treated HUVEC cells was more evident than that of cells treated with free EGCG or just fresh medium (control cells). C57BL/6J black mice with burned corneas were used as a model of neovascularization to test gelatin NPs [95]. Although the authors observed the inhibition of the vascular growth in animals treated with gelatin NPs, the actual mechanism of targeting the vascular cells in a more complicated environment, like in vivo conditions, remains uncertain. They speculate that the residence time of the NPs on the corneal surface is influenced by the attractions of opposite charges between the NPs and the corneal surface. Thanks to this prolonged residence time, the RGD moiety manages to adhere to the damaged area and bind at the receptor [95].

Fangueiro et al. investigated in vivo cationic lipid NPs for the ocular delivery of EGCG [96,97]. The NPs were composed of natural and positively charged lipids and surfactants. Good ocular tolerance of the formulation was observed both in an HET-CAM test, where no sign of vascular response was observed, and in a Draize test, performed on New Zealand white rabbits where no sign of inflammation or excessive lacrimation was observed [96,97].

Luo et al. developed another topical formulation for the delivery of EGCG, consisting of an in situ gelling carrier [98]. A copolymer with gelling properties was synthesized from gelatin and poly(N-isopropylacrylamide). These types of systems make it easier to control the delivery of the active substance. Good biocompatibility and an absence of cytotoxicity were observed when the formulation was tested in HCE-2 cells. Additionally, the gelling system was shown to be effective in the inhibition of the expression of inflammatory cytokines after IL-1β stimulation. Levels of IL-6 and monocyte chemotactic protein-1 (MCP-1) were lower than those in control cells. In vivo studies were performed on DED rabbits, in which DED was induced by topical administration of benzalkonium chloride. After impression cytology conjunctival goblet cells density was assessed through staining with periodic acid-Schiff (PAS). No significant difference in the number of goblet cells were seen in treated rabbits compared to untreated DED rabbits [98].

### 4.4. Curcumin

Curcumin [1,7-bis(4-hydroxy-3-methoxyphenyl)-1,6-heptadiene-3,5-dione] and its health-related properties have been widely reported in many manuscripts of traditional Asiatic medicine [140]. There is a vast number of health benefits that this compound is able to arouse. It is considered to be an efficient agent in aging processes, diabetes, depression, inflammation, cancer, and others [141,142,143]. VCAM-1, MDRP, Bcl-2, ICAM-1, and HIF-1 are just some of the molecular targets of curcumin [144]. Despite all these benefits, curcumin application is restricted by its extensive metabolism and rapid elimination from an organism. It has to be added that additional restrictions are given by a high chemical instability, very low aqueous solubility, and low cellular penetration [145].

In the field of ocular delivery, there have been various approaches to efficiently deliver curcumin to the eye. One such example is the use of the Calix [4] arene derivative, which bears lipid like dodecyl chains [99]. This macrocyclic molecule acts on the principal host-guest, spontaneously self-assembling with curcumin. As reported by the authors, the solubility enhancement of curcumin when complexed was about 9000 times. In addition, the formulation was able to inhibit the nuclear translocation of NF-kB p65 in LPS-stimulated J774A.1 (Mouse BALB/c monocyte macrophage) cells, maintaining levels similar to those in unstimulated control cells. Similar results were observed in the case of iNOS and COX2 factors, which, along with NF-kB, play an important role in starting inflammation [99].

An interesting example of delivery to the posterior segment of the eye is represented by the utilization of two amphiphilic polymers, hydrogenated castor oil 40 (HCO-40) and Octoxynol 40 (OC-40), for the formation of a micellar carrier for curcumin. The micellar formulation containing curcumin was able to decrease the oxidative stress-induced secretion of VEGF in D-407 RPE cells. Additionally, the formulation exhibited sustained release of curcumin over a period of one month [100].

In order to overcome the major disadvantages of topical administration to the eye, Sai et al. investigated the application of an in situ gelling system mixed with micelles. Micelles were formed from PEG-DSPE/Solutol HS 15 (1,2-distearoyl-sn-glycero-3-phosphoethanolamine-N-[methoxy(polyethyleneglycol)-2000]/polyoxyethylene esters of 12-hydroxystearic acid), while the gel-forming part was made from gellan gum, since it possesses a good gelling ability [101]. Permeation enhancers, such as Solutol HS 15, are often included into ophthalmic formulations. These molecules have the ability to improve the permeability of drugs through ocular tissues by modifying the lipidic components of cell membranes or by relaxing the tight junctions associated to the corneal epithelial cells. Except non-ionic surfactants (e.g., Solutol HS 15, polyoxyl 15 hydroxystearate), chelating agents such as EDTA or cyclodextrins are often use for the same purpose [146].

The formulation strategy used by Sai et al. has been shown to be effective in improving not only the solubility of curcumin but also its chemical stability. In 24 h, only 1.4% of formulated curcumin suffered from degradation, while 34% of free curcumin deteriorated. In cellular uptake studies, coumarin-6 was used as a fluorescent probe. The results clearly revealed a development of a higher intracellular green fluorescence from formulated coumarin-6 compared to the one that was in free form. This confirms that an improved ocular retention and absorption was achieved. After the administration of the formulation to rabbits in vivo, no sign of ocular irritation was observed. Additionally, the histopathological analysis of rabbit eyes exposed to the formulation showed no morphological changes in the cornea and conjunctiva [101].

Another example of in situ forming gels is represented by combining Pluronic F127 and Pluronic F68. These two polymers are frequently used for preparing gel matrices. In this case, the sol-gel transition occurs under a thermal stimulus, so these matrices are called thermoresponsive gels. The gel matrix was combined with curcumin-loaded albumin nanoparticles. The formulation was marked as safe after testing for ocular irritation in vivo in rabbits since no signs of injuries were detected in their eyes. Additionally, the concentration of curcumin that was observed in the aqueous humor of treated animals was 5.6-fold higher in the formulation than in free form [102].

### 4.5. Catechin

Catechin is ascribed with antitumoral, antioxidant, and antiviral properties, among others. [147]. The potential application of catechin in ophthalmology was suggested by Lee et al. [148], who proposed the use of this polyphenolic compound in the treatment of cataracts. The results revealed that catechin was able to influence the expression of proteins that are crucial in the regulation of apoptosis in the lens epithelium in a dose-dependent manner. The levels of the pro-apoptotic factor Bax were increased after cataract induction by N-methyl-N-nitrosourea (MNU). Bax levels decreased when catechin was administered to the animals, while the levels of the anti-apoptotic factor Bcl-2 slightly increased [148].

The formation of a complex between PEG and catechin increases the water solubility of the polyphenolic compound at least 100-fold. This formulation strategy exploits the formation of hydrogen between the ether oxygen present in the PEG polymeric chain and the catechol group that is part of the structure of catechin. Catechin formulated in this way was tested as a potential treatment for DED in NOD.B10-H2b mice exposed to desiccating stress to develop experimental DED [103]. The complex PEG/catechin topically administered was able to prevent conjunctival goblet cells from deterioration, as demonstrated by the number of goblet cells recovered in DED-treated animals compared to untreated control animals. The effectiveness of the formulation was further confirmed by tissue immunostaining. Reduced expression of different inflammatory markers (VCAM-1, IL-6, ICAM-1, and TNF-α) was observed in tissues from diseased animals treated with the PEG/catechin complex, indicating an anti-inflammatory effect [103].

### 4.6. Naringenin

Naringenin (4,5,7-trihydroxy-flavanone) is a flavonoid, sub-class of flavanones, and has been ascribed with anti-oxidative, anti-inflammatory, anti-cancer, neuroprotective, and anti-diabetic effects [149,150].

The ocular application of naringenin is limited by its poor solubility, so Zhang et al. [104] developed a carrier that consisted of an inclusion complex between naringenin and sulfolbutylether-β-cyclodextrin (SBE-β-cyclodextrin), aimed to improve flavonoid’s solubility. As a strategy to mask the negative charges of cyclodextrin, the positively charged biopolymer chitosan was added to the existing formulation. Derived from the shells of crustaceans, chitosan is a biocompatible and versatile polymer that possesses low cytotoxicity and is, therefore, suitable for different biomedical applications [151,152]. In addition to the increased solubility of naringenin, naringenin-cyclodextrin/chitosan NPs (Nag-CD/CS-NPs) showed a slower release when compared to the drug complexed with the cyclodextrin only. The formulation was well-tolerated by rabbit eyes in an ocular irritation test. When administered through the Nag-CD/CS-NPs, naringenin had a 4.6-fold higher concentration in the aqueous humor of rabbits compared to the control formulation (suspension of naringenin). The study indicated that such NPs could be suitable for the ocular delivery of naringenin, since the formulation was not irritating, and a prolonged residence time and a higher bioavailability of the drug were achieved [104].

An eye drop formulation containing naringenin with hydroxypropyl-β-cyclodextrin (HP-β-Cyclodextrin), polycarbophil and poloxamer 407 was aimed at treating of the degeneration of retinal neurons caused by AMD. The quantity (C_max_) of naringenin found in the retina was 1927.08–660.77 ng/g at 0.083 h (the collection time of the first sample), which was higher than what was found in the aqueous humor (103.89–20.92 ng/mL) at the same time point. The reason for this probably lies in the combination of excipients used in the eyedrops. HP-β-Cyclodextrin makes naringenin more available on the surface, while polycarbophil, thanks to its mucoadhesive properties, enhances the residence time [105].

### 4.7. Cyanidin

Anthocyanins, like all flavonoids, possess a three-ring structure core, which has, as substituents, different hydroxyl groups that vary in number and position on the core structure. These structural differences have an impact on the biological activity of the anthocyanins [153,154,155].

Among anthocyanins, cyanidin and its glycosides have gained attention because of their pharmacological activities. There has been an increasing number of studies evaluating the combination of these natural compounds with chemotherapeutic agents, as a strategy to relieve or ameliorate side effects related to chemotherapeutic treatments [156]. It is important to underline that cyanidin and its related molecules have also been shown to play a role in the antioxidant protection mechanism in rat hepatocyte Clone 9 cells through the Nrf2-ARE molecular pathway [157].

N-trimethyl chitosan (TMC), a highly soluble derivative of chitosan, was used to decorate the surface of the cyanidine-3-glucoside containing liposomes. The positively charged liposomes were intended to be used as treatment for cataracts in a selenite-induced cataract model in rat pups. Levels of antioxidant enzymes in the group that received the formulation were close to those of the control group in which there was no cataract inducement, indicating the effective delivery of the polyphenol [106].

### 4.8. Myricetin

Myricetin (3,5,7,3′,4′,5′-hexahydroxyflavone) was firstly isolated in the late 18th century in *Myrica nagi*, a plant harvested in India. It is found both in free and glycosylated forms, usually with rhamnose, galactose, or arabinose. Similar to other polyphenolic compounds, myricetin is able to downregulate the expression of inflammatory modulators by inhibiting STAT1 or NF-kB pathways [158].

It also shows activity towards a diverse range of nuclear enzymes such as DNA and RNA polymerase, telomerases, and helicases [159]. Moreover, myricetin is able to reduce the antimicrobial activity of some foodborne pathogens (*Salmonella paratyphi*, *Escherichia coli*, and *Salmonella enteritidis)* [160]. This reduction may include disruption of the membrane of the pathogens or the inhibition of the synthesis of nucleic acids or cell envelope synthesis [161].

Yang et al. have conducted a study where myricetin was used as a treatment for open angle glaucoma. They showed that myricetin was effective in the decrease of ROS levels in vitro, and in restoring SOD levels, thus demonstrating an ability to reduce the oxidative stress that is reported as being critical to the rise of IOP in this type of glaucoma [162,163].

Unfortunately, further applications of myricetin in ophthalmology are limited due to its low solubility and stability. One study tried to address this problem by the encapsulation of myricetin in micelles made from polyoxyl 15 hydroxystearate (HS15) [107]. The authors revealed that the employment of HS15 in the formulation of myricetin improved the solubility of this phenolic compound by at least 656 times. In addition, micellar formulation was able to confer chemical stability at a slightly acidic pH. The in vivo data showed that the drug delivery system was well-tolerated after administration to healthy rabbits. The magnitude of the anti-inflammatory capacity of the HS15 formulation was comparable to that of conventional diclofenac sodium eyedrops, and thus, higher than that of a free myricetin solution [107].

As an alternative to HS15, the same authors reported that PVCL-PVA-PEG, an amphiphilic polymer, possesses exceptional ocular biocompatibility. Besides a solubility enhancement, PVCL-PVA-PEG displayed a greater stabilization of myricetin than HS15. This might be attributed to the extremely low critical micellar concentration of the PVCL-PVA-PEG (3.68 × 10^−8^ M). Both micellar systems show a very high yield of myricetin encapsulation inside the hydrophobic core [108].

### 4.9. Kaempferol

Many polyphenolic compounds have recently been in the central point of research interest because of their proven benefit for human health. One of these is Kaempferol (3,4′,5,7-tetrahydroxyflavone), a low molecular weight flavonol [164].

In a recent study, Du et al. reported that kaempferol has a protective effect on ARPE-19 RPE cells exposed to oxidative stress. This protective effect is exhibited through an antiapoptotic effect and by the downregulation of VEGF expression [165]. Another study that was conducted on Human Retinal Endothelial Cells (HREC) confirmed that kaempferol can be used as a potential treatment for diabetic retinopathy, the angiogenesis that characterizes the disease was suppressed by the inhibition of the glucose-induced expression of PI3K and by the inactivation of Akt1, ERK1/2, and Src [166].

Chuang et al. proposed an ophthalmic formulation of kaempferol based on gelatin NPs for the treatment of corneal neovascularization [109]. Corneal neovascularization implies the formation of new blood vessels in the cornea as a consequence of a corneal damage, inflammation or microbial infections [167]. To improve the stability and the rigidity of the NPs, they were cross-linked with glutaraldehyde. The results showed that the formulation was able to decrease the growth of new blood vessels in the cornea and was effective in the inhibition of cell migration [109].

Another example of a topical ophthalmic formulation with kaempferol was described by Zhang et al. [110]. Polyvinylpyrrolidone (PVP) was employed as a carrier. PVP has a high aqueous solubility and a high ability to complex with poorly water-soluble drugs. The preparation of the nanocomplex between kaempferol and PVP follows a simple procedure by the re-hydration of the thin film formed upon the removal of the organic solvent, where the components of the nanocomplex were dissolved. In accordance with the cytotoxicity tests, which marked the nanocomplex as safe in HCEC, no sign of inflammation, redness, or irritation was observed when the ocular tolerability was assessed in rabbits. The formulation revealed dose-dependent anti-inflammatory activity in vivo after the evaluation of clinical signs of inflammation under a slit lamp [110].

### 4.10. Hesperetin

Results from clinical and pre-clinical studies encourage the use of flavanones for the prevention and cure of pathologies related to the cardiocirculatory system [168]. Additionally, they have been associated with a lower incidence of neurodegenerative diseases [169]. One of the well-studied flavanones is hesperetin (4′-methoxy-3′,5,7-trihydroxyflavanone). Hesperetin also has a potential to lower low density-lipoprotein cholesterol in the plasma and to lower cancer growth, by influencing cell proliferation and migration [170,171]. By inhibition of the COX-2 pathway, hesperetin is also ascribed with anti-inflammatory properties [172].

Hesperetin and its glycosylated form, hesperidin, have displayed potential therapeutic activity in the recuperation of retinal activity lost after retinal ischemia. In addition to the antioxidant and anti-inflammatory activity, both compounds have been found to impede the expression of PECAM-1 and HIF-1α. The two factors play an important role in the mechanisms of angiogenesis and vasculogenesis [173,174]. Considering this, hesperetin and hesperidin could be suitable candidates for the treatment of pathologies affecting the posterior segment of the eye, such as DR or diabetic macular edema. The topical ocular route of administration of hesperetin and hesperidin was evaluated by Srirangam et al. [111]. Due to the low solubility of the two compounds [175], HP-β-cyclodextrin and the randomly methylated beta-cyclodextrin (RM-β-cyclodextrin) were used as a delivery strategy. According to the results, hesperetin showed greater ocular in vivo permeability than hesperidin. For example, the hesperetin concentration found in the cornea 1 h after administration was 15.53 (μg/g of tissue), while the hesperidin concentration was 4.33. The same trend was observed in the retina, in which hesperetin concentration reached 2.62 (μg/g of tissue), while the hesperidin concentration was 1.08. This trend can be explained by the physico-chemical nature of the two compounds; hesperetin has a lower molecular weight than hesperidin and is also more lipophilic. After topical administration to the eye, the molecules are preferentially absorbed by a transcorneal route that favors the absorption of more lipophilic compounds [111].

An attractive formulation for the delivery of hesperetin involves the use of a polymer matrix made from polyethylene glycol N10 (PolyOx^®^ WSR N-10). After mixing the polymer and the polyphenolic compound, the mixture was pressed with a die to obtain a film-like flat structure. Following the topical instillation and upon contact with tears, the polymer film was transformed into a gel-like structure and slowly was washed away from the ocular surface. Based on analysis of the ocular tissues, a notable amount of hesperetin could reach the posterior eye structures. One hour after film instillation containing 10% w/w hesperetin, polyphenol levels were 14.1 μg/gin the cornea and 7.8 and 5.1 μg/g in the retina and sclera, respectively. For the formulation containing 20% w/w hesperetin, the concentrations were higher: 33.5 μg/g in the cornea and 23.5 and 53.2 for the retina and sclera, respectively. This suggests that hesperetin penetrates the eye structures through a transscleral pathway in a dose-dependent manner. Histological analysis of rabbit corneas showed that the formulations did not cause any tissue impairment when compared to controls with untreated corneas [112].

## 5. Conclusions

Thanks to their beneficial effects towards a wide range of diseases, plant polyphenols are an appealing class of compounds. This review evinces their potential application in the treatment of different ocular pathologies. Poor physico-chemical characteristics (such as low solubility in aqueous solvents or low stability) that characterize different compounds from this group limit their further application as therapeutic agents. The solution for their broader employment in the treatment of ocular pathologies lies in the utilization of advanced delivery systems. These systems can be “custom-made” in the base of the disease that is intended to be treated. This means designing a formulation in a manner so that it can exploit a certain peculiarity of the disease (the expression of molecules or alternation of the physiological structure). Thus, problems related to the characteristics of the compound as well as the problems that can be related to the site of delivery can be overcome.

This review shows that polyphenols are interesting in terms of the development of potential therapeutic approaches for the eye. They have shown promising results in disease models of both anterior and posterior segments of the eye. However, these results are mostly limited to in vitro and in vivo models. Therefore, more effort should be placed in consolidating the efficacy of polyphenol-based formulations in clinical studies.

## Figures and Tables

**Table 1:** Summary of different drug delivery systems that were used to deliver polyphenols to the eye.

Polyphenol	Type of Formulation	Pathology	Size (nm)	Entrapment Efficacy	*ζ* Potential (mV)	In Vitro/In Vivo Results	Reference
Resveratrol	Gold NPs	DR	10	N/A	N/A	Reduced retinal expression of VEGF-1, ICAM-1, IL-6, and IL-1β in diabetic rats	[90]
	Nanogel based on chitosan with a high molecular weight	Diseases of the posterior segment	144	59 ± 1	+32 ± 1	No cytotoxicity in ARPE-19 cells; no significant production of IL-6 and IL-8 after an inflammatory stimulus	[91]
	PLGA NPs	AMD	102.7 ± 2.8	65.2 ± 2.2	−47.3 ± 0.9	No cytotoxicity in ARPE-19 cells; reduction of VEGF secretion in the same cell line	[92]
Quercetin alone and in combination with resveratrol	β-cyclodextrin	Dry eye disease	N/A	N/A	N/A	Decrease of the clinical sign and inflammatory response in a murine model of DED	[93]
EGCG	Gelatin NPs coated with hyaluronic acid	Dry eye disease	253.4 ± 7.3	97.8 ± 0.5	+9.2 ± 1.8	No cytotoxicity in Human Corneal Epithelial Cells/improvement of tear secretion and reduced levels of inflammatory cytokines in rabbits with DED	[94]
	Gelatin NPs coated with HA and RGD sequence	Corneal neovascularization	168.8 ± 22.5	97.1 ± 0.55	+19.7 ± 2.0	Inhibition of HUVEC migration rate/inhibition of vessel formation in mice with corneal NV	[95]
	Lipid NPs	Various ocular diseases	183.9 ± 0.6	98.9 ± 0.1	+28.8 ± 0.8	Good ocular tolerance in HET-CAM test/no signs of irritability in NZW rabbits	[96,97]
	In situ gelling system made from gelatin-g-poly(N-isopropylacrylamid)	Dry eye disease	N/A	N/A	N/A	Good biocompatibility, no cytotoxicity, ↓ of the expression of inflammatory cytokines in HCE cells/improved corneal thickness in DED rabbit models	[98]
Curcumin	Derivative of calix [4] arene	Uveitis	82	N/A	+24.3	↓ clinical inflammatory score; inflammatory cytokines in rats	[99]
	Nanomicellar formulation (hydrogenated castor oil-40 and octoxynol-40)	AMD	17.9	82.6 ± 0.5	Slightly-	Doses of 5–10 μM show no cytotoxicity in D407 cells; inhibition of VEGF production under oxidative stress	[100]
	PEG-DSPE/Solutol HS 15 with gellan gum	Diseases related to the ocular surface	13.4 ± 0.1	97.2 ± 2.4	−4.6 ± 0.3	No ocular irritation and no changes in the appearance of the cornea, iris and conjunctiva were observed in NZW rabbits; additionally, ocular retention ↑	[101]
	Gel matrix made from Pluronic F127 and Pluronic F68 in combination with albumin NPs	DR	221.2	85.4 ± 1	N/A	Nonirritating, ↑ corneal permeation, ↑ aqueous humor concentration with respect to the control in NZW rabbits	[102]
Catechin	Complex with PEG	DED	two distinct size distribution of ~5 and ~200 nm	N/A	N/A	Recovery of the density of goblet cells in DED induced NOD.B10-H2b mice; repression of different anti-inflammatory indicators	[103]
Naringenin	SBE-β-CD/Chitosan NPs	AMD	446.4 ± 112.8	N/A	+22.5 ± 4.9	Nonirritating to NZW rabbits’ eyes; after topical application ↑ concentration was achieved in aqueous humor than with the control form.	[104]
	Eyedrop formulation containing HP-β-CD, poloxamer 407, polycarbophil, disodium edentate, BAK, sodium chloride	AMD/retinitis pigmentosa	N/A	N/A	N/A	Consistent quantity of drug was found in the posterior part of the eye after topical administration in NZW rabbits	[105]
Cyanidin	N-trimethyl chitosan (TMC) decorated liposome	Cataract	158.3 ± 2.8	53.7 ± 0.2	+31.7 ± 1	↑ corneal residence time and permeation/restoration of the levels of antioxidant enzymes in cataract induced Sprague Dawley rats	[106]
Myricetin	polyoxyl 15 hydroxystearate micelles	Ocular anti-inflammatory treatment	12.1 ± 0.7	96.1 ± 0.3	−4.2 ± 0.4	Good tolerability in healthy rabbits; decent anti-inflammatory activity	[107]
	PVCL–PVA–PEG micelles	Ocular anti-inflammatory treatment	60.7 ± 1	99.5 ± 0.5	−2.2 ± 0.3	No cytotoxicity observed in HCECs cells/good corneal permeation in NZ albino rabbits; dosage-related anti-inflammatory activity was observed	[108]
Kaempferol	Gelatin NPs cross-linked with glutaraldehyde	CNV	85 ± 8	95± 1	+25.6 ± 2.1	Inhibition of cell migration in HUVECs cells/↓ of the growth of corneal blood vessels in mice with CNV	[109]
	PVP nanocomplex	Various ocular diseases	8.6	93.10	−5.31 ± 0.2	Nontoxic to HCECs cells/good ocular tolerability and anti-inflammatory activity was observed in NZW rabbits	[110]
Hesperetin	hydroxylpropyl beta-cyclodextrin (HP-β-CD), randomly methylated beta-cyclodextrin	Diabetic retinopathy and diabetic macular edema	N/A	N/A	N/A	A significant concentration of the drug was observed in ocular tissues after topical administration in NZW rabbits	[111]
	Film matrix made from PolyOx^®^ WSR N-10	Posterior segment diseases	N/A	N/A	N/A	No damage on corneal tissues was observed; significant levels of the drug after topical instillation detected in the ocular tissue of NZW rabbits	[112]

Note: NZW—New Zealand White rabbits; PLGA—poly(lactic-co-glycolic acid); PVP—polyvinylpyrrolidone; CNV—corneal neovascularization; PVCL-PVA-PEG—polyvinyl caprolactam–polyvinyl acetate–polyethylene glycol; SBE-β-CD—sulphobutylether-βcyclodextrin; PEG-DSPE—1,2-Distearoyl-sn-glycero-3-phosphoethanolamine Poly(ethylene glycol); HA—hyaluronic acid; RGD—tripeptide Arg-Gly-Asp; ↑—increase; ↓—decrease; N/A—not available.

## Data Availability

No new data were created or analyzed in this study. Data sharing is not applicable to this article.

## Abbreviations

AP-1	Activator protein-1
ATP	Adenosine triphosphate
AGEs	Advanced glycation end products
AMD	Age-Related Macular Degeneration
HCE	Corneal epithelial cells
CNV	Corneal neovascularization
COX-2	Cyclooxygenase-2
JNK	c-Jun N-terminal kinases
DR	Diabetic retinopathy
DED	Dry Eye Disease
EGCG	Epigallocatechin gallate
ERK	Extracellular signal-regulated kinases
HREC	Human Retinal Endothelial Cells
HUVEC	Human umbilical vein endothelial cells
HA	Hyaluronic acid
HP-β-Cyclodextrin	Hydroxypropyl-β-cyclodextrin
HIF-1	Hypoxia inducible factor-1
IOP	Increased intraocular pressure
iNOS	Inducible nitric oxide synthase
ICAM-1	Intercellular adhesion molecule 1
IP-10	Interferon gamma-induced protein 10
IL-1	Interleukin-1
IL-1RA	Interleukin-1 receptor antagonist
IL-5	Interleukin-5
IL-6	Interleukin-6
JAK	Janus-activated kinases
LPS	Lipopolysaccharide
LFA-1	Lymphocyte function-associated antigen
MAPK	Mitogen-activated kinases
MDRP	Multi drug resistance protein
Nox	NADPH oxidases
NZW	New Zealand White rabbits
MNU	N-methyl-N-nitrosourea
TMC	N-trimethyl chitosan
NPs	Nanoparticles
NO	Nitric oxide
NOS	Nitric oxide synthases
Nrf2	Nuclear factor erythroid-2 related factor 2
NFAT	Nuclear factor of activated T cells
NF-Kb	Nuclear factor kappa-light-chain-enhancer of activated B cells
PEG	Poly(ethylene glycol)
PEG-DSPE	1,2-Distearoyl-sn-glycero-3-phosphoethanolamine Poly(ethylene glycol)
PVCL-PVA-PEG	Polyvinyl caprolactam–polyvinyl acetate–polyethylene glycol
PVP	Polyvinylpyrrolidone
PKC	Protein kinase C
RM-β-cyclodextrin	Randomly methylated beta-cyclodextrin
RNS	Reactive nitrogen species
ROS	Reactive oxygen species
RPE	Retinal pigment epithelium
SBE-β-cyclodextrin	Sulfolbutylether-β-cyclodextrin
SOD	Superoxide dismutase
TNF-α	Tumor necrosis factor alpha
VEGF	Vascular endothelial growth factor
VCAM-1	Vascular Cell Adhesion Molecule
